# A novel mechanism of ERK1/2 regulation in smooth muscle involving acetylation of the ERK1/2 scaffold IQGAP1

**DOI:** 10.1038/s41598-017-09434-4

**Published:** 2017-08-24

**Authors:** Susanne Vetterkind, Qian Qian Lin, Kathleen G. Morgan

**Affiliations:** Department of Health Sciences, Boston University, 635 Commonwealth Ave., Boston, 02215 USA

## Abstract

Ceramide, a bioactive lipid and signaling molecule associated with cardiovascular disease, is known to activate extracellular signal regulated kinases 1 and 2 (ERK1/2). Here, we determined that the effect of ceramide on ERK1/2 is mediated by ceramide signaling on an ERK scaffold protein, IQ motif containing GTPase activating protein 1 (IQGAP1). Experiments were performed with aortic smooth muscle cells using inhibitor screening, small interfering RNA (siRNA), immunoprecipitation (IP), immunoblots and bioinformatics. We report here that C6 ceramide increases serum-stimulated ERK1/2 activation in a manner dependent on the ERK1/2 scaffold IQGAP1. C6 ceramide increases IQGAP1 protein levels by preventing its cleavage. Bioinformatic analysis of the IQGAP1 amino acid sequence revealed potential cleavage sites for proteases of the proprotein convertase family that match the cleavage products. These potential cleavage sites overlap with known motifs for lysine acetylation. Deacetylase inhibitor treatment increased IQGAP1 acetylation and reduced IQGAP1 cleavage. These data are consistent with a model in which IQGAP1 cleavage is regulated by acetylation of the cleavage sites. Activation of ERK1/2 by ceramide, known to increase lysine acetylation, appears to be mediated by acetylation-dependent stabilization of IQGAP1. This novel mechanism could open new possibilities for therapeutic intervention in cardiovascular diseases.

## Introduction

In vascular smooth muscle, ERK1/2 activation can lead either to contraction, as in a healthy blood vessel, or proliferation, which is associated with cardiovascular disease. We have shown previously that the outcome of ERK1/2 activation in vascular smooth muscle cells is dependent on the type of stimulus used to activate the kinase^1^. It is well known that scaffold proteins are critical in assembling stimulus-specific pathways of ERK1/2 activation^2^. We have previously demonstrated that two scaffold proteins, caveolin-1 and IQGAP1, together assemble a signaling cascade connecting activation of protein kinase C to activation of a sub-fraction of cellular ERK1/2 associated with actin in smooth muscle cells^3^.

Activation of sphingomyelinase generates ceramide, which is a bioactive lipid and signaling molecule present in atherosclerotic plaques. Ceramide is known to play a role in oxidized LDL-induced cell proliferation and arteriosclerosis^4, 5^. Ceramide can either increase proliferation or induce apoptosis^6, 7^. Ceramide is also known to activate ERK1/2 via the ERK1/2 scaffold KSR1 kinase suppressor of Ras (KSR1), also known as ceramide activated kinase in mammalian cells^8, 9^ but only a single study has linked KSR to vascular smooth muscle cells and only in diabetes^10^. Since our previous work has demonstrated a role for IQGAP1 in ERK1/2 signaling^1, 3^, the goal of the present study was to test the hypothesis that ceramide might modulate ERK1/2 signaling by an effect on ERK1/2 scaffolds and IQGAP1 in particular.

## Results

### Ceramide amplifies ERK1/2 activation in response to serum but not in response to a phorbol ester

**Figure 1:**
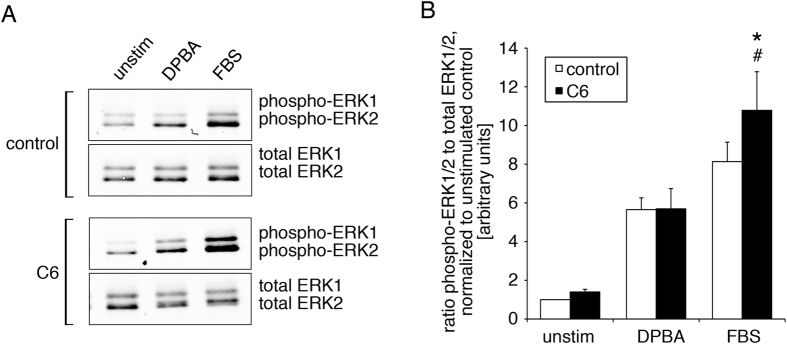
Ceramide increases serum-induced ERK1/2 phosphorylation. Aortic smooth muscle cells were treated with ceramide C6 for 6 hours; control cells were treated with diluent alone. Cells were stimulated with either 12-deoxyphorbol 13-isobutylate 20-acetate (DPBA) or serum (FBS) for 5 minutes, or left unstimulated. Cell lysates were analyzed by western blotting and densitometry. (**A**) Typical immunoblots (cropped) show ceramide enhanced the increases in ERK1/2 phosphorylation after FBS, but not after DPBA stimulation. (**B**) Statistical analysis of 7 independent experiments. *control + FBS versus C6 + FBS: p = 0.024, # C6 + DPBA vs. C6 + FBS: p = 0.025.

We have previously shown that ERK1/2 activation in smooth muscle cells can be either proliferative or contractile in its outcome, depending on the stimulus, and that the different outcomes of stimulation are determined by ERK1/2 scaffolds^1, 3^. The question arises, then as to whether ceramide exerts effects on smooth muscle proliferative signaling by targeting specific ERK1/2 signaling pathways. To this end, we treated aortic smooth muscle cells with the cell permeable C6 ceramide, and analyzed ERK1/2 phosphorylation as a measure of ERK1/2 activation in response to either a phorbol ester, 12-deoxyphorbol 13-isobutylate 20-acetate (DPBA), a stimulus that activates contractile pathways in vascular smooth muscle, or fetal bovine serum (FBS), a stimulus that activates proliferative pathways. Whereas ceramide treatment had no significant effect on phorbol ester mediated activation of ERK1/2, the relative amount of phosphorylated ERK1/2 in response to serum was significantly increased after ceramide treatment compared to control treated cells (Fig. 1).

### IQGAP1, but not KSR1, is involved in ceramide-mediated amplification of ERK1/2 activation

Based on our previous studies^1, 3^, demonstrating stimulus-specific and scaffold-specific ERK1/2 signaling pathways in aortic smooth muscle cells, we speculated that the stimulus-specific effect of ceramide on ERK1/2 activation might be mediated by an ERK1/2 scaffold.

**Figure 2:**
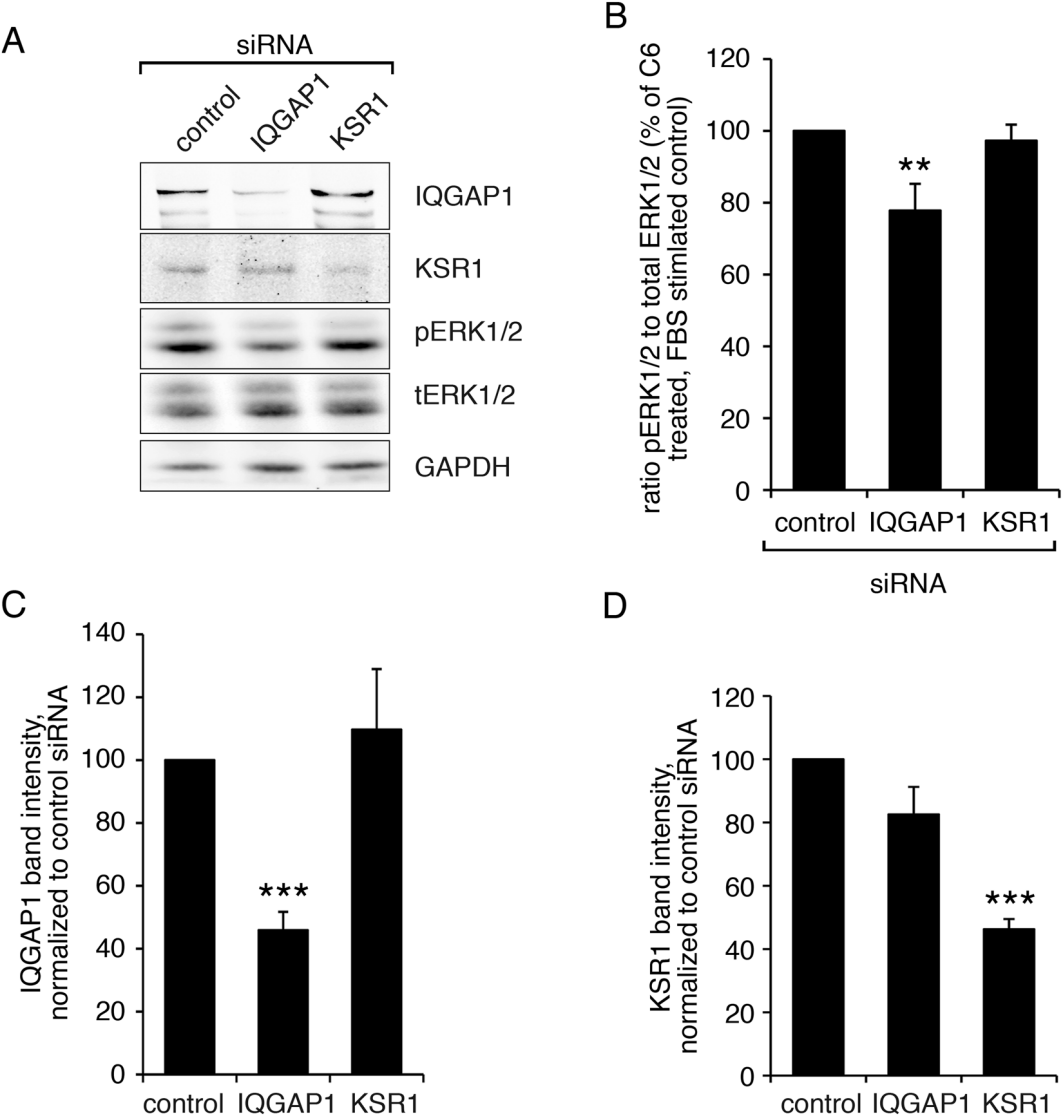
SiRNA knockdown demonstrates a role for IQGAP1 in ceramide-induced ERK1/2 activation. (**A**) Aortic smooth muscle cells were treated with control, IQGAP1, or KSR1 siRNA. 5 days after transfection, cells were treated with ceramide (6 hours, 50 µg/ml). Additionally, cells were stimulated with FBS (10%) for 5 minutes before preparation of cell extracts. Cell lysates were analyzed for expression of IQGAP1, KSR1, phospho-ERK1/2 and total ERK1/2 using specific antibodies. GAPDH staining is shown as reference. Cropped gels are shown. (**B**) The statistical analysis of 5 independent experiments shows a significant decrease of relative ERK1/2 phosphorylation after knock down of IQGAP, but not KSR1. Statistical analysis of the degree of siRNA-induced knock down are shown in (**C**) for IQGAP1 and in (**D**) for KSR1.

KSR1 is an ERK1/2 scaffold reported to be involved in ceramide signaling pathways^9, 11, 12^ in other systems. Thus, we investigated a possible role of KSR in the effect of ceramide in aortic smooth muscle cells (Fig. 2). However, siRNA against KSR1 decreased KSR1 levels (Fig. 2A,D) but did not significantly affect ERK1/2 phosphorylation in the presence of C6 plus FBS (Fig. 2B).

IQGAP1 is another well known ERK scaffolding protein that we have previously shown to play a role in these aortic smooth muscle cells^3^. Knockdown of IQGAP protein levels with siRNA (Fig. 2A,C), in contrast to the results for KSR1, significantly reduced C6 plus FBS-induced ERK1/2 phosphorylation (Fig. 2B). This indicated a role for IQGAP1, but not KSR1, in the ceramide mediated increase in ERK1/2 phosphorylation in aortic smooth muscle cells and raised the question of the mechanism involved.

### Ceramide stabilizes IQGAP1 by preventing its proteolytic processing

**Figure 3:**
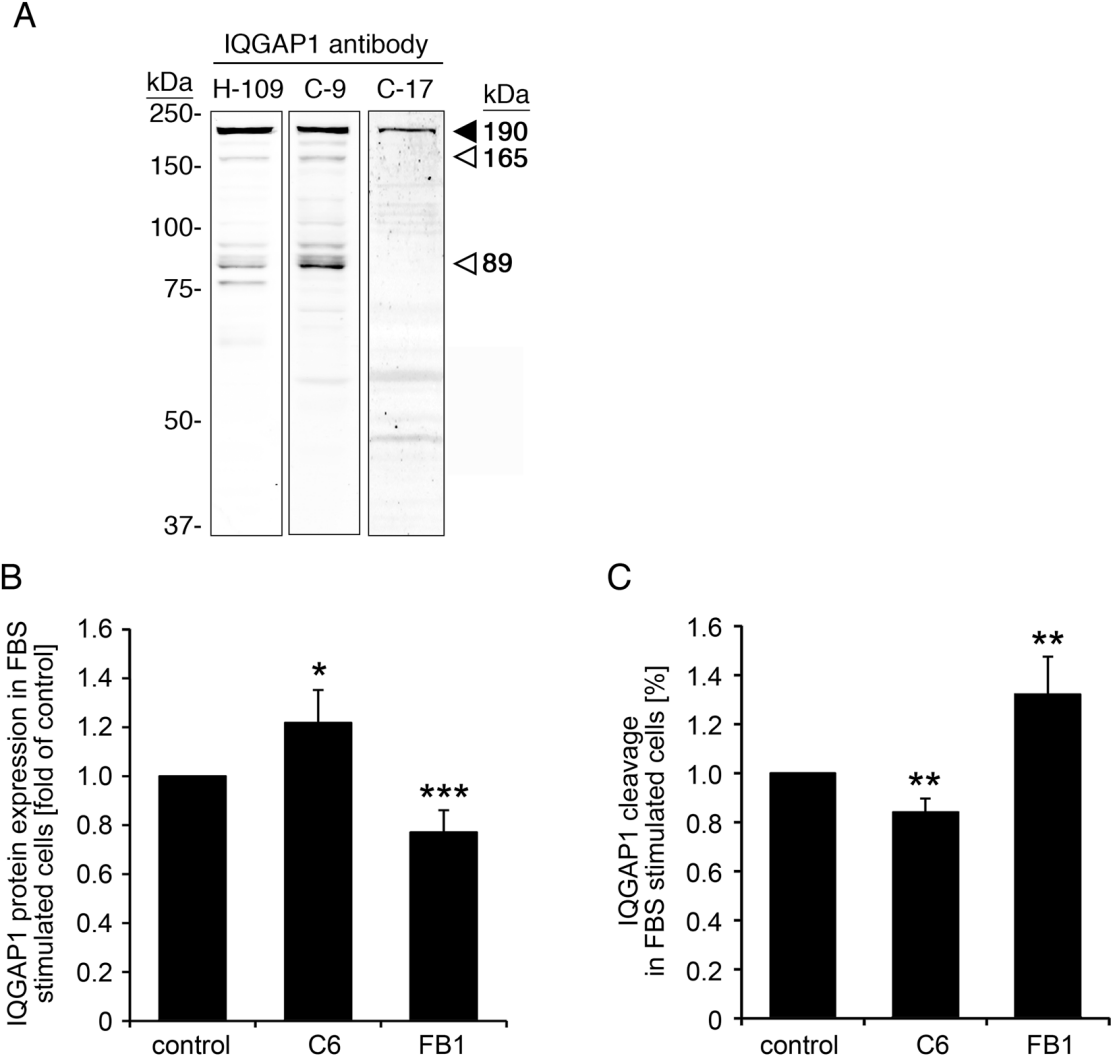
Ceramide stabilizes IQGAP1 by inhibiting its cleavage. (**A**) IQGAP1 antibodies raised against the N-terminal part of the protein (C-9 and H-109) show the same pattern of additional bands, whereas an antibody raised against the C-terminal region of IQGAP1 does not detect these bands. (**B**,**C**) VSM cells were treated with either C6 ceramide (50 µmol/L, 6 hours) or the ceramide synthase inhibitor, fumonisin B1 (15 µM, 24 hours) or control treated. Cells were stimulated with serum (10% FBS, 5 minutes) before preparation of whole cell lysates and western blot analysis. H109 was used to detect IQGAP1. (**B**) Expression levels of full length IQGAP1 were analyzed by normalization of the full length IQGAP1 band to GAPDH. Normalized full length IQGAP1 was then normalized to the untreated control. (**C**) Cleaved fragments of IQGAP1 are shown as a percentage of total IQGAP1 (=sum of full length IQGAP1 plus cleaved fragments) normalized to the untreated control.

After immunoblotting with an IQGAP1-specific antibody that recognizes an epitope in the N-terminal region of IQGAP1 (rabbit polyclonal anti-IQGAP1, H-109, Santa Cruz), we observed additional bands of 185 kDa(faintly), 165 kDa and 89 kDa in addition to full length IQGAP1 (190 kDa) (Fig. 3A). The intensities of these bands are reduced along with the intensity of full length IQGAP1 after siRNA knock down of IQGAP1 as shown for some of the bands in Fig. 2A, indicating that these bands are specifically stained by the IQGAP1 antibody and might represent splice variants or cleavage products of IQGAP1. However, we were unable to find any reports on IQGAP1 splice variants in the literature. The additional bands were detected by using a different antibody that is raised against amino acids 3–37 (mouse monoclonal anti-IQGAP1, clone C-9, Santa Cruz), but not by an antibody that was raised against the C-terminus of IQGAP1 (goat polyclonal anti-IQGAP1, clone C-17, Santa Cruz) (Fig. 3A), consistent with the additional bands being products that arise from cleavage in the C-terminal region of the protein.

Interestingly, when we analyzed the amount of full length IQGAP1 in FBS-stimulated samples (Fig. 3B), we detected (with H-109) a significant increase in IQGAP protein expression in lysates from cells that had been pretreated with ceramide, whereas the ceramide synthase inhibitor fuminisin B1 (FB1) had the opposite effect and led to reduced full length IQGAP1 levels. Conversely, when we quantitated the total amount of the putative IQGAP1 cleavage fragments and expressed this as a percentage of the total IQGAP1 band staining (Fig. 3C), we found decreased IQGAP1 cleavage in samples that had been treated with ceramide, but increased cleavage of IQGAP1 in cells treated with the ceramide synthesis inhibitor FB1. Thus ceramide appears to stabilize IQGAP1 by preventing its cleavage.

### IQGAP1 sequence analysis reveals acetylation motifs close to predicted cleavage sites

**Figure 4:**
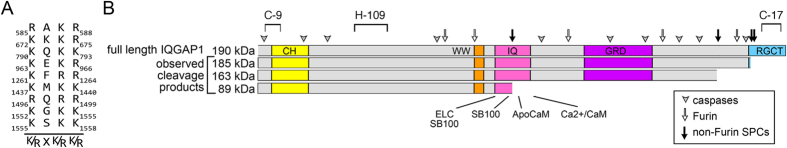
Observed IQGAP1 cleavage products in comparison to positioning of SPC and caspase cleavage sites. (**A**) List of K/R-X-K/R-K/R motifs found in the IQGAP1 amino acid sequence. (**B**) Diagram to show full length IQGAP1 and its putative fragments. The epitopes recognized by the different antibodies used in Fig. 3 are indicated in the top row. Fragment lengths were calculated based on SDS-PAGE with a molecular weight marker (Precision Plus, BioRad) as standard. The positions of SPC cleavage motifs are indicated by black and white arrows, with the white arrows also matching furin cleavage sites. For comparison, predicted caspase cleavage sites are also shown (white arrowheads). Abbreviations: CH, calponin homology; WW, poly-proline protein-protein interaction domain; IQ, IQ motifs; GRD, GAP (GTPase activating protein) Related Domain; RGCT, RasGAP-C-terminus IQGAP1. Domain map modified after^30^.

To narrow down the possible proteases responsible for IQGAP1 cleavage, we screened the IQGAP1 amino acid sequence for possible cleavage sites using protease cleavage prediction software (Expasy PeptideCutter). Predicted cleavage sites were then compared to the experimentally found cleavage products, but none of the proteases considered by this tool produced a cleavage pattern similar to the observed band pattern in our experiments.However, analysis of the IQGAP1 amino acid sequence for recurring motifs did reveal the motif K/R-X-K/R-K/R, that is present in rat IQGAP1 with 9 repeats. This motif is similar to the consensus motif for Subtilisin/kexin Proprotein Convertases (SPCs), also named Paired basic Amino acid Cleaving Enzymes (PACEs), namely K/R-X_n_-K/R↓, with the downward arrow indicating the position of the cleavage site within or relative to the motif^13^. Some copies of this motif also conform with the more specific consensus cleavage site recognized by furin (Fig. 4B), a member of the SPC family, which consists of the sequence R-X-K/R-R↓^14^. The positioning of those SPC motif copies that have a lysine in position P1 (the residue closest to the predicted cleavage site) matches closely with the expected cleavage sites derived from calculated fragment sizes of IQGAP1 (black arrows in Fig. 4B). While they match the general consensus for SPCs, these sites are unlikely to be cleaved by furin, which requires an arginine in position P1^14, 15^.

Interestingly, in addition to being putative cleavage sites, these motifs also resemble a type of lysine acetylation motif with the consensus K-X-K-K^16^ (Fig. 4A). Lysine acetylation has been shown to regulate protein stability, for example in the cases of beta secretase BACE1^17^, FoxA2^18^ and glutamase carboxypeptidase II^19^. The overlap of acetylation motifs in close vicinity of the putative cleavage sites therefore raises the possibility that IQGAP1 cleavage could be regulated by acetylation.

### Acetylation regulates IQGAP1 protein stability

**Figure 5:**
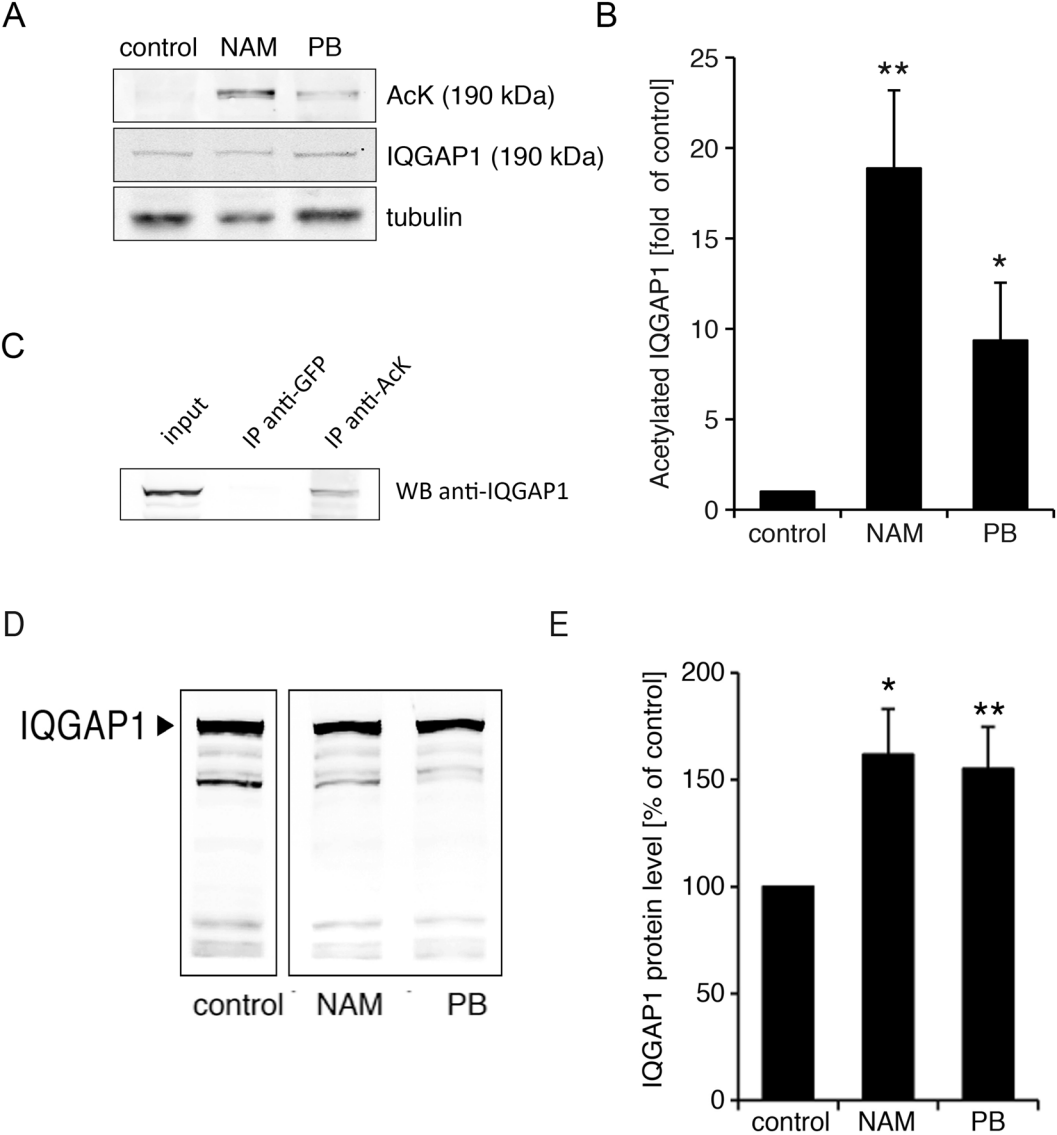
Acetylation and cleavage of IQGAP1. (**A**) Cells were treated with nicotinamide (NAM) or phenylbutyrate (PB) at 5 mmol/l for 24 hours. Cell lysates were then analyzed by western blotting. Acetylated proteins were detected with an acetylated lysine-specific antibody. Identity of the IQGAP1 band was confirmed by subsequent co-staining with an anti-IQGAP1 antibody. Cropped gels are shown. (**B**) Bar graph and statistical analysis of IQGAP1 acetylation in untreated, NAM treated and PB treated cells. (**C**) Endogenous IQGAP1 is immunoprecipitated from lysates of NAM-treated aortic smooth muscle cells with an acetylated lysine-specific antibody, but not with a GFP antibody. A cropped gel is shown. (**D**) Treatment with NAM or PB results in reduced cleavage of IQGAP1 relative to full length IQGAP1. Cropped gels are shown. (**E**) The bar graph shows full length IQGAP1 relative to the sum of full length IQGAP1 and smaller IQGAP1 fragments (normalized to control). (*p < 0.05, **p < 0.01).

To determine whether IQGAP1 is acetylated/deacetylated, we treated aortic smooth muscle cells with the histone deacetylase (HDAC) inhibitors sodium phenylbutyrate (PB), which inhibits class I and class II HDACs, or nicotinamide (NAM), which inhibits the class III HDACs, also called the Sirtuins. Cell lysates were then analyzed for lysine acetylation using a lysine acetylation-specific antibody. After HDAC inhibitor treatment, several bands showed increased signal intensity. Staining of the same membranes with an anti-IQGAP1 antibody revealed an overlap of the 190 kDa acetylated lysine band with IQGAP1 (Fig. 5A). The bar graph in Fig. 5B shows the statistical analysis of p190 IQGAP1 acetylation in untreated, NAM treated and PB treated cells. Statistically significant increases were seen with both deacetylase inhibitors. Moreover, IQGAP1 was pulled down from NAM-treated aortic smooth muscle lysates in IP experiments with an acetylated lysine-specific antibody (Fig. 5C).

Cleavage of IQGAP was noted to be less prominent in the presence of the deacetylase inhibitors NAM and PB (Fig. 5D). When analyzed by densitometry, quantitating the amount of full length IQGAP, under control conditions, or in the presence of NAM, or PB, and expressing those values as a percentage of the control demonstrated a statically significant inhibition of cleavage, and a relative increase in full length IQGAP was seen with both HDAC inhibitors (Fig. 5E).

### Does ceramide increase IQGAP1 acetylation?

**Figure 6:**
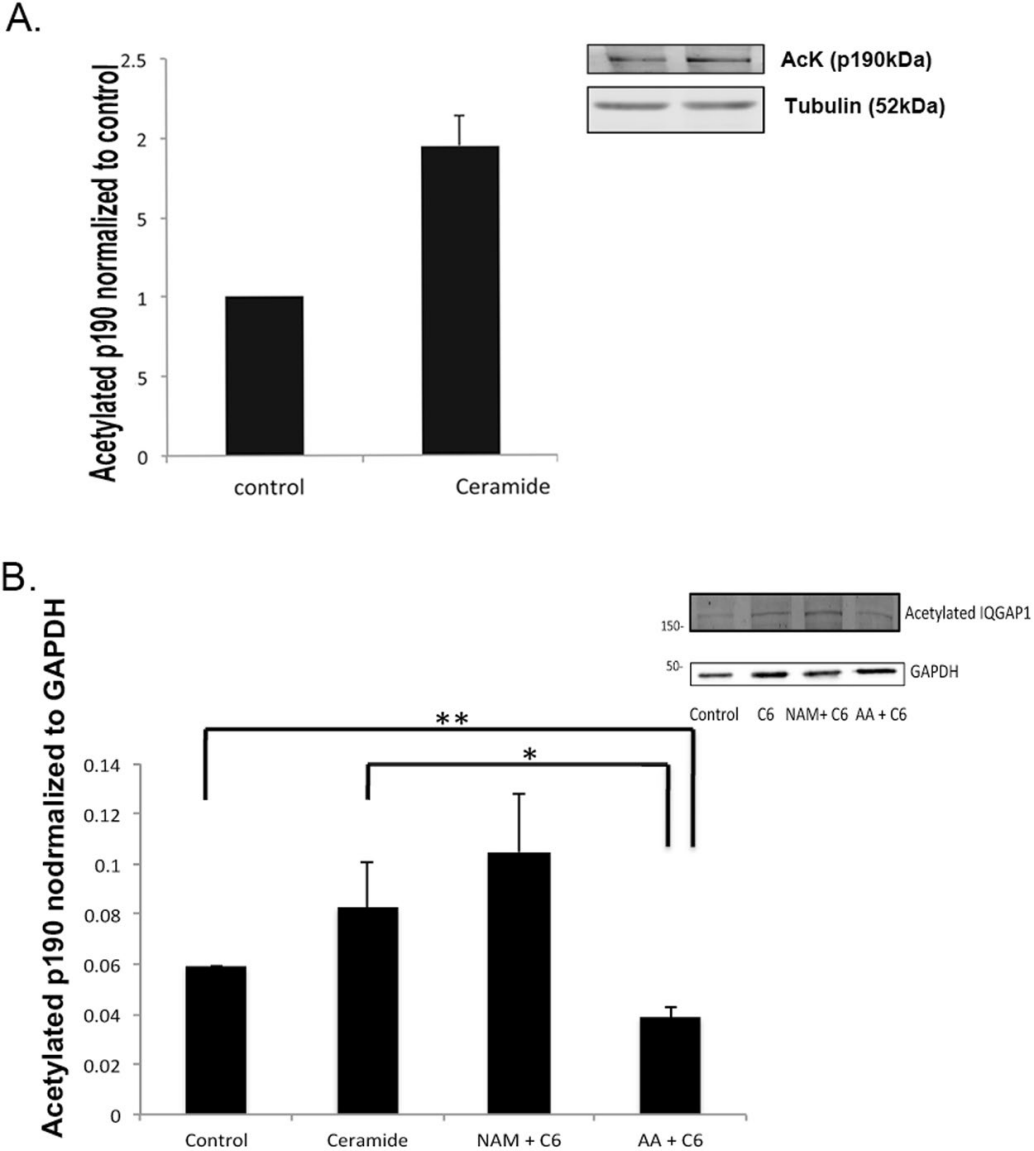
Ceramide increases acetylation of IQGAP. (**A**) Ceramide (6 hr) increases acetylation of band co-migrating with IQGAP. Inset: Typical blot. (**B**) Ceramide increases acetylation by activating HATs. Treatment (1 hour) with the HAT inhibitor anacardic acid (AA) inhibits effect of ceramide to increase acetylation. NAM (1 hour) has no significant additional effect of ceramide to increase acetylation. (inset) Typical blot. Cropped gels are shown.

Ceramide has been shown to promote lysine acetylation^17, 20^. Since HDAC inhibitor treatment and ceramide have similar effects on IQGAP1 stability, the question arises as to whether the effect of ceramide on IQGAP1 is mediated by increased acetylation of IQGAP1. If so, we should be able to detect an increased acetylation in the p190 band. As is shown in Fig. 6A, ceramide does indeed increase acetylation of a 190 kDa protein consistent with acetylation of IQGAP1.

The increased acetylation could be due to either inhibition of HDACs or to activation of histone acetyltransferases (HATs). To determine which mechanism is involved, we used the HDAC inhibitor, NAM to determine if blocking HDACs can produce any additional effect to that of ceramide. If ceramide acts through inhibition of HDACs no additional effect is expected. Alternatively, if ceramide increases acetylation by activating HATs, a HAT inhibitor should prevent the effect of ceramide. As is shown in Fig. 6B, the HDAC inhibitor NAM did not produce a statistically significant additional effect on acetylation. In contrast, the HAT inhibitor had a dramatic effect to decrease acetylation. Thus, the data are consistent with ceramide increasing acetylation by activating HATs.

### HDAC inhibitor treatment increases ERK1/2 activation

**Figure 7:**
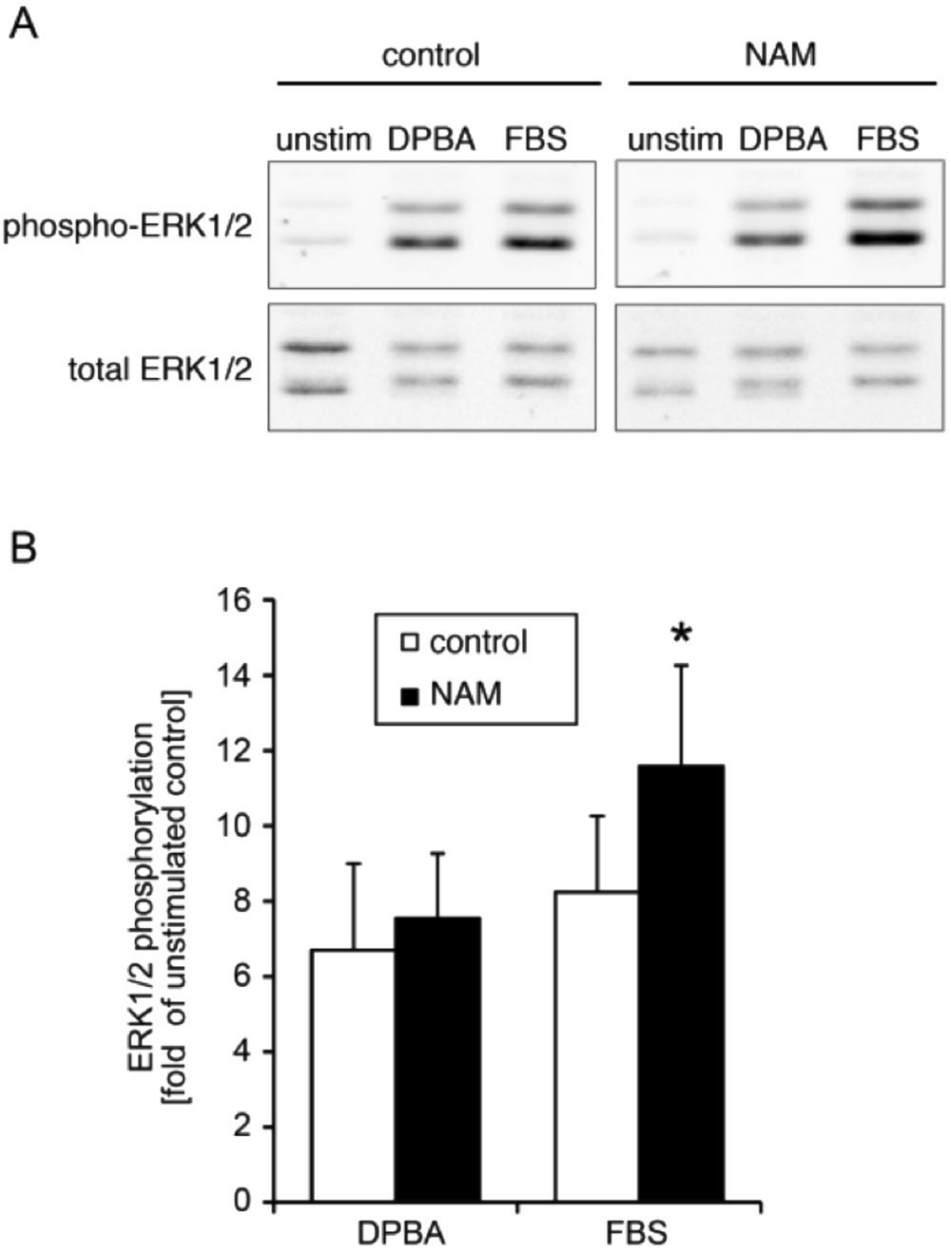
Effect of HDAC Inhibitor NAM on ERK1/2 activation with a proliferative versus contractile stimulus. (**A**) Typical blots. Cropped gels are shown. (**B**) Increases in ERK1/2 phosphorylation with and without NAM for DPBA versus FBS (*p < 0.05).

Since IQGAP is an ERK scaffold, and we have shown that ceramide increases ERK activation (Fig. 1), the question arises as to whether increasing acetylation of IQGAP with NAM would also alter ERK phosphorylation levels. Thus, we used FBS to trigger a proliferative ERK activation pathway and found (Fig. 7A,B) that a significant augmentation of ERK activation occurred. Interestingly, phorbol ester, a contractile rather than a proliferative stimulus, also increased ERK activity but this was not significantly augmented by NAM, consistent with a difference in the scaffolding.

## Discussion

Our data are consistent with ceramide, which we show here increases lysine acetylation, stabilizing IQGAP1 by increasing its acetylation, thereby regulating ERK1/2 activation.

**Figure 8:**
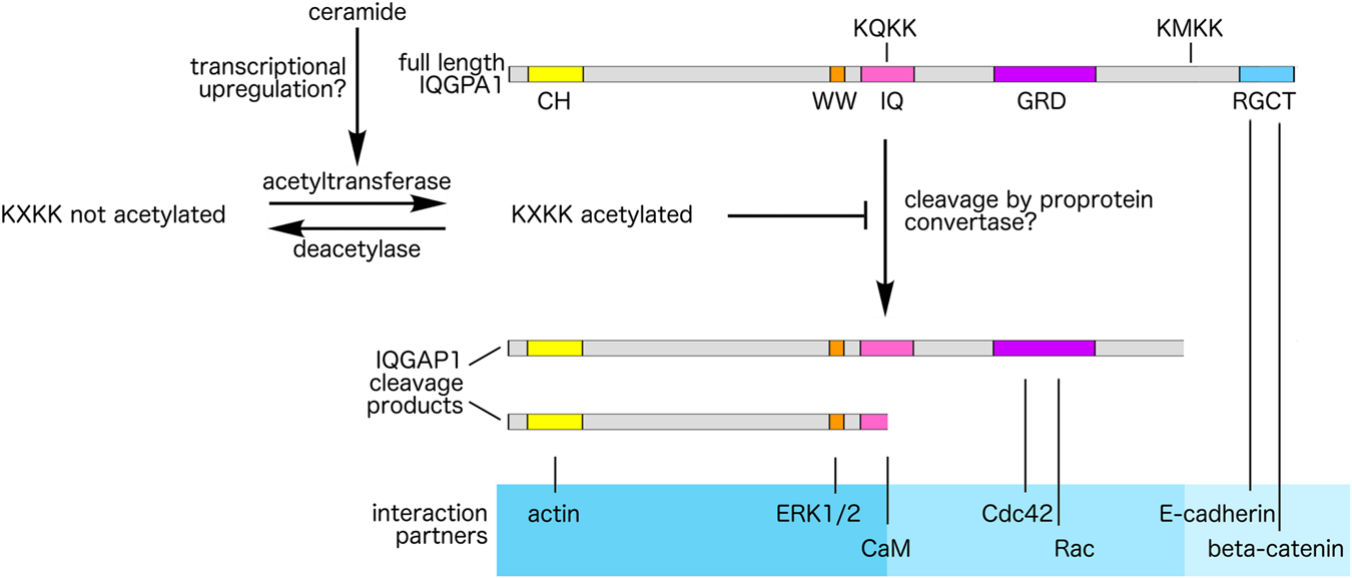
Model of the regulation of cleavage by acetylation in the presence of ceramide. Based on our data, we suggest a model in which IQGAP1 cleavage is regulated by acetylation at or in close proximity to cleavage sites, and in which ceramide stabilizes IQGAP1 by increasing acetylation and thus, reducing IQGAP1 cleavage. Two cleavage sites that overlap with acetylation motifs are indicated as KQKK and KMKK above the full length IQGAP1 domain map. The domain composition of the resulting IQGAP1 fragments is expected to affect their spectrum of binding partners (shown in the blue-shaded box) and thus, scaffold function. CH, calponin homology (actin binding); WW, proline-rich protein protein interaction domain (ERK1/2 binding); IQ, IQ domain (calmodulin binding); GRD, GTPase activating protein related domain (Cdc42 and Rac binding); RGCT, RasGAP-C-Terminus (beta catenin and E-cadherin binding).

Furthermore, based on our data, we suggest a model (Fig. 8) in which cleavage of IQGAP1 is regulated by its acetylation at or in close proximity to the cleavage sites. Others have reported similar ceramide-induced acetylation and stabilization of tubulin^20^ as well as the secretase BACE1 which affects stability of the Alzheimer’s disease (APP)^17^. In the case of BACE1, the stabilizing effect of acetylation has been attributed to ceramide-induced transcriptional upregulation of two acetyltransferases^21^. Our findings that ceramide-induced IQGAP1 acetylation is caused by HAT activation rather than HDAC inhibition, and that the effect of ceramide required a treatment duration of six hours, indicate the possibility that IQGAP1 stabilization, too, is based on ceramide-induced transcriptional upregulation of acetyltransferase genes.

Because of the modular structure of IQGAP1, cleavage could also generate interesting IQGAP1 fragments that may have specific functions in signaling. Cleavage of IQGAP1 may lead to the generation of smaller IQGAP1 fragments with specific function due to specific subdomain composition (e.g. removal of C-terminal cadherin binding site). Interestingly, the potential SPC cleavage sites are all outside of the IQGAP1 functional domains (see domain map in Fig. 4), with the exception of one site in the IQ domain. Cleavage of this site could generate a cleavage products with either the first or the last two IQ motifs only. Of note, the four IQ motifs show selective binding to myosin essential light chain, the glial calcium binding protein S100B, apocalmodulin and Ca^2+^/calmodulin^13, 22^. Furthermore, smaller IQGAP1 fragments containing only one binding domain might by themselves modulate cellular signaling by acting as decoy peptides.

The acetylation motif KXKK, that appears to play a role in IQGAP1 stability, is typical for steroid receptors, e.g. the glucocorticoid receptor. It may seem surprising that IQGAP1 harbors this motif although it is itself not a steroid receptor; however, it was recently shown to interact with a steroid receptor^23^. None of the previously reported IQGAP1 acetylation sites overlap with the KXKK motifs. 10 acetylation sites were identified by Chen *et al*.^24^, and 8 sites were shown to be acetylated by Lundby *et al*.^25^. However, between the two studies, only 3 sites were detected in both screens, indicating that in each screen many acetylation sites might be missed.

Our results also highlight the importance of carefully considering experimental conditions in the interpretation of scaffold function. For example, we have shown previously that IQGAP1 is required for phorbol ester induced ERK1/2 activation^3^. However, no effect of HDAC treatment on phorbol ester induced ERK1/2 activation was observed in the present study. This apparent contradiction can be explained by the fact that although IQGAP1 is required, its acetylation does not play a role in phorbol ester-induced ERK activation

In conclusion, our data are consistent with a model where activation of ERK1/2 by ceramide is mediated by ceramide-induced lysine acetylation close to proteolytic cleavage sites of the ERK1/2 scaffold IQGAP1 and consequent stabilization IQGAP. The data also indicate that ceramide-induced acetylation of IQGAP1 occurs via the activation of Histone acetyl transferases. This novel mechanism could open new possibilities for selective therapeutic intervention in cardiovascular diseases. For example, if ceramide-dependent activation of ERK through acetylation of the scaffold IQGAP leads to cardiovascular disease, the present results suggest that it would be possible to leave intact other ERK scaffolds while inhibiting the acetylation pathway to selectively inhibit the ceramide pathway. A similar strategy has been used in the cancer field to cause apoptosis but not necrosis by selective inhibition of p38 MAPK but not JNK or ERK leading to selective cancer cell cytoxicity^26^.

## Methods

### Reagents and antibodies

General laboratory reagents were purchased from Sigma (St. Louis, MO) and BioRad (Hercules, CA) and were of analytical grade or better. Fetal bovine serum (FBS, Invitrogen, Carlsbad, CA) for cell culture and for stimulation was used at 10%. For phorbol ester stimulation, 12-deoxyphorbol 13-isobutyrate 20-acetate (DPBA) was used at 3 µmol/L. The duration of stimulation with FBS or DPBA was 5 minutes. C6 ceramide (Sigma) was used at 50 µmol/L for 6 hours. 0.1% alcohol, used to dissolve ceramide, was used alone as a vehicle control. Ceramide synthase was inhibited by treatment with fumonisin B1 (Cayman Chemical, Ann Arbor, MI) at 15 µM for 24 hours. Dimethylsulfoxide, ethanol, and/or methanol were used as control treatment as appropriate. The Sirtuin inhibitor nicotinamide (NAM, Sigma) and the class I and II histone deacetylase (HDAC) inhibitor sodium phenylbutyrate (PB, Sigma) were both used at 5 mmol/L for 24 hours. The histone acetyltransferase inhibitor anacardic acid (Milipore Sigma, Billerrica, MA) was used at 10 µM for 1 hour. For western blots, the following primary antibodies were used: rabbit polyclonal anti-pERK1/2 (1:2000, Cell Signaling, Danvers, MA), mouse monoclonal anti-ERK1/2 (1:500, Cell Signaling), mouse monoclonal anti-KSR1 (1:100, BD Biosciences, San Diego, CA), rabbit polyclonal anti-IQGAP1 (1:1000, Santa Cruz Biotechnology, Santa Cruz, CA), mouse monoclonal anti-IQGAP1 (1:1000, Santa Cruz), goat polyclonal anti-IQGAP1 (1:500, Santa Cruz), rabbit polyclonal anti-glyceraldehyde 3-phosphate dehydrogenase (GAPDH) antibody (1:200,000, Sigma), rabbit polyclonal anti-acetylated lysine antibody (1:300, Cell Signaling), mouse monoclonal anti-tubulin (1:3000, Sigma). As secondary antibodies, IRDye (R) 680 or IRDye (R) 800 CW labeled goat anti-rabbit, donkey anti-goat or goat anti-mouse IgGs were used (1:1000, LI-COR).

### Cell culture and siRNA transfection

A7r5 rat aorta cells (ATCC, Manassas, VA) were cultured as described previously^3^. For all experiments, cells were subjected to serum starvation (0% serum for 24 hours) to ensure differentiation to the smooth muscle-like phenotype^27, 28^. SiRNA knock down of IQGAP1 and KSR1 as well as control siRNA treatment was performed as described previously^3^ using lipofectamine 2000 (Invitrogen). Cells were processed for experiments 5 days after siRNA transfection.

### Cell extracts and IP experiments

For preparation of whole cell lysates, plates were washed three times with ice-cold phosphate-buffered saline (pH 7.2), drained on ice and then scraped off in lysis buffer (140 mmol/L NaCl, 3 mmol/L MgCl_2_, 1 mmol/L dithiothreitiol and 0.5% Nonidet-P40 in a 20 mmol/L sodium phosphate buffer, pH 8.0) or, for IP experiments, in IP lysis buffer (50 mmol/L NaCl, 10% glycerol, 1% Nonidet-P40 in a 10 mmol/L sodium phosphate buffer, pH 8.0). Lysis buffers were supplemented with protease inhibitors. Cell lysis was performed on ice for 30 minutes. Lysates were subsequently cleared by centrifugation (16,000 rcf for 10 minutes at 4 °C). For IP experiments, lysates in IP lysis buffer were incubated rotating at 4 °C over night in the presence of anti-acetylated lysine antibody (5 μl antibody for 500 mg total protein) crosslinked to Protein G-dynabeads (R) (Invitrogen). For control IPs, rabbit anti-GFP antibody (Clontech, Mountain View, CA) crosslinked to protein G-dynabeads (R) was used. The immobilized antigen-antibody complexes were washed three times with IP lysis buffer. Bound proteins were eluted from the immobilized antibodies with sodium dodecylsulfate (SDS) sample buffer and further processed for western blotting.

### Western blot

Proteins in IP samples or whole cell lysates were separated by SDS-polyacrylamide gel electrophoresis (PAGE) according to standard procedures. For immunoblots, proteins were transferred from SDS gels to nitrocellulose membranes (Whatman, Florham Park, NJ) and stained with specific primary antibodies and appropriate secondary antibodies. Bands were detected using an Odyssey(R) infrared scanner a system that is highly linear in the range of intensities used. Odyssey 2.1 software was used for densitometric analysis of the raw data. For comparison of protein expression levels, bands were normalized to either GAPDH or tubulin signals on the same membrane. To determine relative ERK1/2 phosphorylation, pERK1/2 signals were normalized to the total ERK1/2 signal on the same membrane.

### Statistics and sequence analysis

All values given in the text are means ± standard error. Statistical significance was evaluated using two-tailed Student’s t-tests, and differences with p values below 0.05 were considered significant. Data from at least 5 independent experiments were used for statistical analyses. For IQGAP1 sequence analysis, the PeptideCutter software^29^, which can be found at http://web.expasy.org/peptide_cutter, was used to identify potential caspase cleavage sites. Potential proprotein convertase cleavage sites were located by sequence comparison with the known proprotein converatase motifs^15^.

### Data availability

All data generated or analysed during this study are included in this published article.

## References

[1] Vetterkind S, Saphirstein RJ, Morgan KG (2012). Stimulus-specific activation and actin dependency of distinct, spatially separated ERK1/2 fractions in A7r5 smooth muscle cells. PLoS One.

[2] Morrison DK, Davis RJ (2003). Regulation of MAP kinase signaling modules by scaffold proteins in mammals. Annu Rev Cell Dev Biol.

[3] Vetterkind S, Poythress RH, Lin QQ, Morgan KG (2013). Hierarchical scaffolding of an ERK1/2 activation pathway. Cell Commun Signal.

[4] Auge N (1996). The sphingomyelin-ceramide signaling pathway is involved in oxidized low density lipoprotein-induced cell proliferation. J Biol Chem.

[5] Witztum JL, Steinberg D (1991). Role of oxidized low density lipoprotein in atherogenesis. J Clin Invest.

[6] Li X, Becker KA, Zhang Y (2010). Ceramide in redox signaling and cardiovascular diseases. Cell Physiol Biochem.

[7] Petrache I, Petrusca DN, Bowler RP, Kamocki K (2011). Involvement of ceramide in cell death responses in the pulmonary circulation. Proc Am Thorac Soc.

[8] Yao B (1995). Phosphorylation of Raf by ceramide-activated protein kinase. Nature.

[9] Zhang Y (1997). Kinase suppressor of Ras is ceramide-activated protein kinase. Cell.

[10] Nemoto S, Taguchi K, Matsumoto T, Kamata K, Kobayashi T (2012). Pravastatin normalizes ET-1-induced contraction in the aorta of type 2 diabetic OLETF rats by suppressing the KSR1/ERK complex. Am J Physiol Heart Circ Physiol.

[11] Yu W, Fantl WJ, Harrowe G, Williams LT (1998). Regulation of the MAP kinase pathway by mammalian Ksr through direct interaction with MEK and ERK. Curr Biol.

[12] Roy F, Therrien M (2002). MAP kinase module: the Ksr connection. Curr Biol.

[13] Seidah NG, Chretien M (1999). Proprotein and prohormone convertases: a family of subtilases generating diverse bioactive polypeptides. Brain Res.

[14] Nakayama K (1997). Furin: a mammalian subtilisin/Kex2p-like endoprotease involved in processing of a wide variety of precursor proteins. Biochem J.

[15] Duckert P, Brunak S, Blom N (2004). Prediction of proprotein convertase cleavage sites. Protein Eng Des Sel.

[16] Faus H, Haendler B (2006). Post-translational modifications of steroid receptors. Biomed Pharmacother.

[17] Costantini C, Ko MH, Jonas MC, Puglielli L (2007). A reversible form of lysine acetylation in the ER and Golgi lumen controls the molecular stabilization of BACE1. Biochem J.

[18] van Gent R (2014). SIRT1 mediates FOXA2 breakdown by deacetylation in a nutrient-dependent manner. PLoS One.

[19] Choi JY, Kim JH, Jo SA (2014). Acetylation regulates the stability of glutamate carboxypeptidase II protein in human astrocytes. Biochem Biophys Res Commun.

[20] Zhu QY (2011). C6-ceramide synergistically potentiates the anti-tumor effects of histone deacetylase inhibitors via AKT dephosphorylation and alpha-tubulin hyperacetylation both *in vitro* and *in vivo*. Cell Death Dis.

[21] Ko MH, Puglielli L (2009). Two endoplasmic reticulum (ER)/ER Golgi intermediate compartment-based lysine acetyltransferases post-translationally regulate BACE1 levels. J Biol Chem.

[22] Pathmanathan S (2008). IQ motif selectivity in human IQGAP1: binding of myosin essential light chain and S100B. Mol Cell Biochem.

[23] Erdemir HH, Li Z, Sacks DB (2014). IQGAP1 binds to estrogen receptor-alpha and modulates its function. J Biol Chem.

[24] Chen Y (2012). Quantitative acetylome analysis reveals the roles of SIRT1 in regulating diverse substrates and cellular pathways. Mol Cell Proteomics.

[25] Lundby A (2012). Proteomic analysis of lysine acetylation sites in rat tissues reveals organ specificity and subcellular patterns. Cell Rep.

[26] Kaltenmeier CT (2017). A Tumor Cell-Selective Inhibitor of Mitogen-Activated Protein Kinase Phosphatases Sensitizes Breast Cancer Cells to Lymphokine-Activated Killer Cell Activity. J Pharmacol Exp Ther.

[27] Kimes BW, Brandt BL (1976). Characterization of two putative smooth muscle cell lines from rat thoracic aorta. Exp Cell Res.

[28] Firulli AB (1998). A comparative molecular analysis of four rat smooth muscle cell lines. In Vitro Cell Dev Biol Anim.

[29] Gasteiger J (2006). Chemoinformatics: a new field with a long tradition. Anal Bioanal Chem.

[30] Brown MD, Sacks DB (2006). IQGAP1 in cellular signaling: bridging the GAP. Trends Cell Biol.

